# Anxiety disorders and PTSD in Palestine: a literature review

**DOI:** 10.1186/s12888-020-02911-7

**Published:** 2020-10-16

**Authors:** Mohammad Marie, Sana SaadAdeen, Maher Battat

**Affiliations:** 1 Mental Health at Faculty of Medicine and Health Sciences at An-Najah National University, Nablus, Palestine; 2 Nurse at primary health care centers-Ministry of Health-Palestine, Community Mental Health Nursing program at An-Najah National University, Nablus, Palestine; 3 Adult oncology/Hematology Ward – An-Najah National University Hospital, Nablus, Palestine

**Keywords:** Anxiety disorder, Anxiety, PTSD, Palestine, Gaza strip, West Bank

## Abstract

**Background:**

The WHO reports that anxiety disorders are the most common mental disorders worldwide. Most people who experience such events recover from it; however, people with post-traumatic stress disorder (PTSD) continue to be severely depressed and anxious for several months or even years following the event. Palestinians are particularly at a higher risk for developing anxiety disorders and PTSD due to the continuous exposure to political violence, prolonged displacement, and other limitation on professional, educational, financial opportunities, and mental health services. This paper aims to provide a systematic review of the literature and established studies concerning Anxiety disorders besides PTSD in Palestine.

**Methods:**

PubMed, Science Direct, Google Scholar was used to search for materials for the critical analysis of empirical articles. The following aspects were taken into consideration: study type, sample, and key findings.

**Results:**

In this review, we included about twenty-four studies from Palestine (West Bank and Gaza). Five studies relate to children, five relate to adolescents, three relate to women, three relate to physical diseases, and four relate to gender and age differences. Results show that anxiety disorders and PTSD are one of the most common mental disorders in Palestine. Anxiety and PTSD develop from a complex set of risk factors, including genetics, personality, and life events. They are mostly associated with low quality of life and disability. The results indicate that a significant proportion of Palestinian experiencing serious issues that deal with several challenges, distinct barriers including; inconsistent availability of medications, absence of multidisciplinary teamwork, insufficient specialists, fragmented mental health system, and occupation.

**Conclusion:**

As primary prevention, the occupation has to have considered as the main source of anxiety and other mental health disorders in Palestine. Besides, there is a need to implement a mental health care system through multidisciplinary work and raising awareness regarding the prevalence of mental disorders.

## Background

### Anxiety disorders and PTSD worldwide

Over one billion people globally have one or more mental disorders. The WHO reports that anxiety disorders are the most common mental disorders worldwide [1]. Anxiety disorders are frequent there lifetime prevalence ranging between 5 and 25% of the population, and a 12-month prevalence ranging between 3.3 and 20.4%, world widely [2]. Anxiety disorders are the most common mental disorders globally especially in women more than in men [1]. Similarly, anxiety disorders are the most common mental disorders in the U.S, affecting 18.1% of the population every year. People with an anxiety disorder are six times more likely to be hospitalized for psychiatric disorders than those who do not suffer from anxiety disorders and three to five times more likely to go to the doctor. It is well established that anxiety disorders develop from a complex set of risk factors, including personality, genetics, and life events [3].

Some anxiety disorders, in particular phobias, social anxiety, and separation anxiety have a very early age of onset, range from 5 to 10 years of age. While others (generalized anxiety disorder (GAD), panic disorder (PD), and post-traumatic stress disorder (PTSD)) tend to have a later age-of-onset distribution (median 24–50), with a much wider cross-national variation [2].

Moreover, anxiety disorders are highly treatable, though only 36.9% of those suffering receive treatment. It affects 25.1% of children between 13 and 18 years old. Besides, Researchers found that untreated children with anxiety disorders are at higher risk of being engaged in substance abuse, perform poorly in school, miss out on essential social experiences. In the U.S, 45.9% of women and 65% of men who got raped are likely to develop the disorder [3].

As well as, researchers found lower psychiatric treatment levels in an updated survey for 24 communities in 21 countries from WHO surveys in lower-income countries. Also, 41.3% of those with 12-month anxiety perceived a need for mental health care; only 9.8% received possibly adequate psychiatric treatment. 9.8% had a 12-month anxiety disorder, 27.6% of whom received mental health care and treatment [4].

Because of: their relatively high prevalence, their tendency towards chronicity, and substantial comorbidity, anxiety disorders are associated with significant disability and poor quality of life [5]. Anxiety disorders are also very costly. It has been estimated that the total costs of anxiety disorders were € 74.4 billion for 30 European EU countries in 2010 [6].

The standard Anxiety disorder treatments are First of all, psychotherapy which includes cognitive- behavioral therapy and psychodynamic psychotherapy. Then, the pharmacological treatment which includes Selective serotonin reuptake inhibitors (SSRI), Benzodiazepines, non-addictive anxiolytic, Buspirone, Beta-blocker, Tetracyclic antidepressant (TCAS) [7]. Furthermore, Narrative Exposure Therapy which is a short-term psychological treatment for PTSD that has been investigated in various contexts especially for torture survivors, particularly in the Middle East and North Africa (MENA) region where health systems are unable to meet the increasing needs of mental health disorders caused by war and displacement [8].

However, many barriers limit the effective treatment of anxiety disorders and PTSD. Structural and health system weaknesses, including scarcity mindset as well as lack of awareness and costs of treatment and stigma perceived by the people who experience anxiety disorders, further limit their treatment [9].

### Anxiety disorders in Arab and Muslim countries

Anxiety was discussed by some famous Islamic scholars in ancient history such as al-Razi, Imam Al-Ghazali, Ibn Kathir, Muhammad ‘Uthman Najati, and others [10]. According to Imam Al-Ghazali, anxiety is a mental disease developed by the heart. It grows from an unhealthy soul of a human being. It is similar to other diseases such as anger, hatred, envy, sadness, pride, and others. He characterized anxiety as fear towards certain things which lead to restless and frustrated feelings [11]. Imam Al-Ghazali also discussed several types of anxiety for example the fear of old age, fear of death, fear of Allah, fear of poverty, fear of losing status and jobs, and fear of being different from others. He described all these fears as coming from a peaceful heart when someone does not give full trust in destiny (Qada and Qadar) set by Allah and does not have complete reliance on Allah.

Moreover, According to al-Ghazali, meditation can deepen ‘ma’rifatullah’ (knowing Allah) in the heart which is the beginning of purification in the soul. Prophet Mohamed has been practicing this meditation while he was in the Cave of Hira ‘. Here, he has found a peace that he had never experienced before. He also received the first revelation from Allah [12]. Furthermore, Ahmed ibn Sahl al-Balkhi (m. 934 M)‘s famous book *Masalih al-Abdanwa al-Anfus*has discussed the relationship between body and soul and describes the spiritual and psychological health [11]. Also, Ali al-Tabari (m.923 M), a famous medical practitioner who developed Islamic psychotherapy to heal patients suffering from mental disorders. This has been mentioned in his famous book entitled ‘*Firdaus al*-*Hikmah*’ [13].

On the other hand, the Holy Quran stated among the ways to apply in the psychotherapy process to treat general anxiety disorders through performing Salat (prayer). Through Salat, the person would be able to express all the hope by asking help from Allah during difficult situations. If the Salat is performed sincerely, then it can purify the heart and transform one’s life to be calm, confident, and disciplined [14].

In short, anxiety disorders are generally caused by mental or emotional instability. On the other hand, the Islamic view considered it a soul disease to some extent rather than a mental disorder as promoted by the psychiatrists from the West [15].

The following paragraphs will present some studies conducted in different Arab and surrounding countries regarding anxiety disorders and PTSD. By using a stratified random sampling technique, 1552 adolescent school-age boys and girls in Abha city, southwestern Saudi Arabia, were screened for mental health using an Arabic validated version of SCL-90-R. Their ages ranged between 14 and 19 years. The most frequent mental symptoms were phobic anxiety (17.3%). The researchers found the insignificance prevalence between girls and boys. Some sociodemographic conditions such as father education, mother working status, ranking among brothers and sisters, and type of school were significantly affecting mental health. They also showed secondary school students enrolled in Islamic schools have 1.5 times the risk to have obsessive-compulsive compared to those enrolled in general school. An interaction between genetic and environmental factors might explain the increase in behavior disorders in boys [16].

Also, generalized anxiety disorder (GAD) was found with highly prevalent among children and adolescents. An Egyptian study aimed to explore the prevalence and socio-demographic risk factors related to anxiety disorders especially (GAD) in adolescents and children. The tools which were used in this study: the general health questionnaire (GHQ, 28 items with cut point 14), the anxiety scale; prepared by Castello and Comrey, 1953, the structured clinical interview for those obtained 15 degrees and above in GHQ or 75 degrees and above on anxiety scale, and the Psychiatric disorders evaluation questionnaire. The study was conducted among 1200 students. The sample consisted of 493 who were males and represented 44.7% and 611 who were females and represented 55.3% and their ages ranged from 12 to 18 years. The researchers used different anxiety scale. The researchers found that depression was the most prevalent 23.8% and then anxiety which was 6.69%. According to psychological diagnosis, anxiety disorders were prevalent in the age group from 15 to 16 years and GAD was more common in males. The study also showed that the increased family size leads to increased occurrence of psychological disorders. As well as the dead of the father may lead to the marriage of the mother and consequently the stressors were increased [17].

Moreover, another study conducted using a descriptive cross-sectional design was carried out among high school students in Irbid, Jordan. The study aimed to explore the prevalence of mood and anxiety disorders and to investigate their association with gender and other socio-demographic factors. The sample consisted of 1103 adolescent students. Their ages ranged between 13 and 18 years. More than half of the students were females. The study showed a prevalence of 16.3% for any anxiety disorder. Female adolescents were significantly more likely to have mental disorders than males. Moreover, adolescents who were living with both parents were significantly more likely to have mental disorders than those living with one parent or other people. However, the researchers presented an explanation which might be that when both parents are present, any conflict between them might affect the mental health and anxiety levels of their adolescent offspring [18].

On the other hand, some studies were carried out to explore the prevalence of anxiety disorders and PTSD in particular after being exposed to several traumas such as war and conflict. According to a study conducted by assessing 3048 participants post-conflict communities in Algeria, Cambodia, Ethiopia, and Palestine. PTSD and other anxiety disorders were the most frequent problems and reported most in people who had experience of violence associated with armed conflict. For example, it was associated with higher rates of disorder that ranged from a risk ratio of 2.10 for anxiety in Algeria to 10.03 for PTSD in Palestine [19].

Moreover, Arab immigrant women are vulnerable to posttraumatic stress disorder (PTSD) because of a higher probability of being exposed to war-related violence, and immigration stressors. These findings were showed in a descriptive study conducted among Arab Muslim immigrant women, particularly those from Iraq and Lebanon, who have been exposed to war. The sample consisted of 546 women. All data were collected from face to face interviews by Arab women. They used different measurements of anxiety. The researchers found over a third of the participants (44%) reported living through or witnessing three or more traumatic events. The most commonly reported types were military combat or war zone (88.6%). Also, women who lived in a refugee camp were more at risk of PTSD than other women [20]. Furthermore, in Afghanistan, more than two decades of war which affected negatively women’s freedom of movement, access to healthcare, and education have affected the mental health status. The prevalence of PTSD was higher in women than in men (48% met diagnostic criteria compared to 32%) in data from the Centers for Disease Control and Prevention’s (CDC) 2002 national survey of postwar Afghani mental health [21].

In summary, it was not clear if the prevalence of anxiety disorders and PTSD are similar or not between males and females in different Arab countries. However, it was clear that some multiple sociodemographic conditions and environmental factors significantly play a role in affecting and causing mental health disorders in particular anxiety disorders. Also, anxiety disorders may exhibit high levels of lifetime comorbidity with one of the other disorders, for example, anxiety disorders with Depression. So understanding the underlying causes of these disorders can provide insight into the etiology and inform classification and treatment. Moreover, PTSD was also detected after being exposed to war-related violence in some Arab and Muslim countries which harmed several aspects of life, especially psychological status.

### Mental health care in Palestine

Palestine (the occupied Palestinian territory) that includes the Gaza Strip and West Bank is an eastern Mediterranean country seeking independence and freedom. The social geography of modern Palestine, especially the area west of the Jordan River, has been greatly affected by the dramatic political changes and wars that have brought this small region to the attention of the world [22].

Gaza Strip is a narrow piece of land lying on the coast of the Mediterranean Sea. The West Bank is an area of land between Israel and Jordan. The West Bank and Gaza together constitute Palestine, which is administered by the Palestinian Authority (PA) [22]. Besides, refugees account for 73.1% of the Gaza Strip and 30.2% of West Bank populations [23]. Most of the population is Muslim, and common Palestinian values include strong family bonds, social identity from family and community, and a holistic outlook on life, rootedness to the land [24].

Palestine has been under occupation by Israel since the 1948 war. It was considered by Palestinians as the beginning of ‘Catastrophe’. Palestinians have experienced ongoing suffering, traumas, and social distress [25]. In 1987, the first uprising, known as the Intifada, broke out in Gaza Strip and West Bank. In 2000, Israeli punishment measures and practices were again in place to discontinue the second Intifada. After that, in 2002, Israel started to build a physical barrier with parts of it isolating the Palestinians’ cities and villages. Israel called it a ‘fence’ and Palestinians called it a ‘Separation Wall’ [26]. In 2008, 2012, and 2014, there was a prolonged siege involving movement restrictions on food and individuals, especially in Gaza Strip. Palestinians experienced violations of their human rights, loss of life, harm, and home destruction [27].

The health care system in Palestine is complex and fragmented; basic public health and primary care are offered by four main facilities: The Palestinian Authority (Governmental), the United Nations (United Nation Relief and Work Agency for Palestinians (UNRWA)), non-governmental organizations (NGOs), and private health care services such as pharmacies or clinics [28].

In 2002, the World Health Organization began a Palestine Mental Health Project, in cooperation with the Palestinian Ministry of Health. The WHO has been trying to develop the Palestinian health system and planning for building new community mental health centers [28]. The Gaza Community Mental Health Project (GCMHP) provides community mental health services, trains in community mental health and human rights, and sponsors field research [29]. Nowadays, mental health services in Palestine are community-based care. However, there are only 13 community mental health clinics or centers in West Bank, in addition to one psychiatric hospital in Bethlehem [30].

The developmental challenges of mental health care vary from country to country influenced by their income. Additionally, other factors have determined the efficacy of mental health services including political decisions, social factors, and the kind diversity of cultures [31]. Considering the extremes of war experienced in Palestine over the last 70 years, a mental health system is facing specific challenges linked with occupation and political conflict. Restrictions on freedom and movement considerably limit patients from receiving care outside of their area of residence, and the cost of treatment, and inconsistent availability of medications on the WHO essential medicines list, in addition to insufficient specialists and absence of multidisciplinary teamwork present further access issues [32].

Furthermore, mental disorders in Palestine remain underreported, under-resourced, under-treated, and mental health services underfunded. These services are unable to meet the burden of need. There is a severe lack of human and infrastructure resources, for example, the total number of psychiatrists is 20 in the West Bank [32]. According to the available researches; the mental health problems are generally high in the Palestinian population [24] According to statistics of the Palestinian Center for Counseling, the results of patients who went to psychiatry during the past 3 years (2007, 2008, and 2009) 25% of patients suffer from anxiety disorders [33]. Furthermore, the Community Mental Health Program in Gaza in 2017 reports the percentage of anxiety disorders among patients visiting psychiatric clinics in Gaza 26% [34]

Lack of feeling safe is the main cause of mental disorders, such as anxiety, phobias, depression, and PTSD [35]. Palestinian population especially adolescents and children were constantly exposed to a lack of security and safety due to the impact of the Israeli occupation practices [36]. The percentage of students in public schools in Area (C) which is managed by Israeli military forces suffers from psychological conditions and social difficulties 36.4% in 2011 and 69.4% in 2012 [37].

Anxiety disorders represent 32.9% of health problems that cause the most disability [38]. Furthermore, Anxiety disorders can alter behavior and cognition as well, yet little is known about the particular domains they affect [39]. According to the recent DSM-5 classification, anxiety disorders included generalized anxiety disorder, specific phobia, social anxiety disorder, separation anxiety disorder, agoraphobia, panic disorder, and selective mutism. The symptoms can interfere with daily activities such as job performance, school work, and relationships. They are characterized by excessive worrying, uneasiness, and fear of future events, such that they affect social and occupational functioning [40, 41].

On the other way, most people who experience such events recover from them, but people with PTSD continue to be severely depressed and anxious for months or even years following the event. As well as, The DSM-5 outlines the diagnostic criteria for PTSD as having exposure to the traumatic event, the presence of some specific symptoms, persistence avoidance of stimuli, negative alteration in cognition, mood, arousal, and reactivity associated with the traumatic events [42].

In this study, the authors aimed to summarize the literature about Anxiety disorders and PTSD in Palestine. This is the first review to summarize Anxiety disorder and PTSD in Palestine.

## Methods

In this literature review study, the articles were gathered through intensive searching in the following electronic databases: PubMed, Science Direct, and Google Scholar. The MeSh terms used in the searching process are Anxiety Disorder AND PTSD AND Palestine, Anxiety, AND Gaza Strip OR West Bank. These words were also used to search in the Arabic language to identify articles indexed in An-Najah University Journal for Research which 2 articles translated from Arabic to the English language. More than 24 studies included, following the IMRAD style (Introduction, method, results, and discussion section), in addition to 4 Palestinian statistics used from formal websites. Search history and study filtration show in Appendix A**(**checklist results**).** The full-text articles were critically appraised using the framework of Rees (2003) [43]. Old reviews were also included due to lack of articles regarding this subject considering subjects were not recruited specifically through the mental health system or concentrating on the multifactorial cause of Anxiety disorders rather than the occupational factors to avoid the selection bias. The search results were independently screened and extracted by the three authors, and all discrepancies were resolved by the principal investigator (MM). The study was conducted following the Preferred Reporting Items for Systematic Reviews and Meta-Analyses (PRISMA) guidelines shown in Additional file 1. The inconsistencies are resolved by consensus between authors which include the critical appraisal and the writing up of the sections.

### Inclusion and exclusion criteria

Due to the significant lack of mental health studies in Palestine; all of the related articles about anxiety and PTSD were included in this study. There were also practical difficulties to access to the studies in libraries shelves in East Jerusalem and the Gaza strip because of the presence of military barriers and the Separation Wall.

### Quality assessment

The quality assessment of each study was assessed according to a checklist. The checklist consists of the following items: including clear study aims, adequate sample size, response rate reported, and losses have given, an adequate description of data, appropriate statistical analysis. a representative sample (with justification), clear inclusion and exclusion criteria, valid and reliable measure of mental health, response rate reported, and losses have given, adequate description of data, appropriate statistical analysis. Two investigators (MB and SS) independently assessed article quality, and inconsistencies were resolved by the principal investigator (MM).

### Characteristics of included studies

Searching history shown in Appendix (A) and the characteristics of the included studies are presented in table Appendix B. After reviewing the depth of selected search findings and obtaining necessary data. Twenty-four studies were included in this review conducted in Palestine (West Bank and Gaza Strip). Five studies focused on children, five studies focus on adolescents, three studies focused on women, three studies focused on physical diseased and four studies focused on gender and age differences and other Palestinian statistics including one thesis study. All reviewers independently charted the data, discussed the results, and continuously updated the data-charting form in an iterative process. We grouped the studies by the topic they studied. For the critical analysis of empirical articles, the following aspects were considered: study type, the survey instrument used, aim, sample, and the key findings. A data-charting form was jointly developed by two reviewers to determine which variables to extract.

## Results

### Overview regarding Anxiety disorders and PTSD in Palestine

#### Palestinian children

The influence of the occupation on Palestinian children has been studied. Children are considered to be at risk due to living in camps or exposed to a long term of violence. One of these studies was conducted among 237 children living in the Gaza Strip and was selected randomly from 112 schools. The age ranged between 9 and 12 years. Children completed the revised manifest anxiety scale (a questionnaire with yes/no answers for 28 anxiety items and nine lie items), and teachers completed the Rutter scale. The study found that the Gaza hostilities made landmark traumas on children such as posttraumatic stress disorders and anxiety. Anxiety physiological symptoms reported: Insomnia, nightmares, and sweating. Also, they found that the Inner-city was exposed to anxiety more than villages. This would reflect the effect of social system support from families in rural areas [44].

Furthermore, another study was carried out to explore the prevalence and nature of comorbid post-traumatic stress reactions among 403 Refugee children living in four camps in the Gaza Strip. Measures included a Checklist of Gaza Traumatic Events, the Child Post Traumatic Stress Reaction Index (CPTSD-RI), and the Short Mood and Feelings Questionnaire (MFQ). Many items included within these checklists which cover different types of traumatic events that a child may have been exposed to in the particular circumstances of the occupied region. The study found both CPTSD-RI and MFQ scores were significantly correlated and independently predicted by the number of experienced traumatic events. They concluded that children living in occupation and blockade zones were at high risk of suffering from PTSD [45].

Moreover, a study was carried out to explore the long-term effects of occupation on the Palestinian children in the Gaza Strip. The sample included 1137 children randomly selected from all parts of the Gaza Strip. They completed a Checklist of Traumatic Experiences (CTE), Symptoms of Post-Traumatic Stress Disorder Scale (PTSD), and Personality Assessment Questionnaire (PAQ). The results showed 41% of children suffered (PTSD). Of the 41% of children with PTSD, the levels of symptoms were as follows: 20% of children suffered from an acute level of PTSD, 22% suffered from moderate levels of PTSD, and 58% suffered from low levels of PTSD. The symptoms of PTSD varied in different forms. The first one: *cognitive symptoms* (e.g. a child might take a long time to get to sleep, or cannot stop thinking about the trauma). Second: *emotional symptoms* e.g. easily getting tense and nervous, feeling sad and fearful, bedwetting) [46].

Also, a study was conducted in the Gaza Strip in areas under ongoing shelling, siege, and other acts of military violence. The sample included 100 families, with 200 parents and 197 children. The age ranged between 9 and 18 years Parents and children completed measures of experience of traumatic events (Gaza Traumatic Checklist), PTSD (Children’s Revised Impact of Events Scale, PTSD Checklist for parents), and anxiety (Revised Children’s Manifest Anxiety Scale, and Taylor Manifest Anxiety Scale for parents). Both parents and children reported a high number of experienced traumatic events and high rates of PTSD and anxiety scores [47].

Child labor is another issue in countries with high unemployment rates which may press the children and families to push the children outside the school classes to earn money for the support of the children and their families. A study was conducted to establish an association between labor-related variables and child mental health problems. The data were collected over 2 months during the ongoing occupation and siege. The sample consisted of 780 children who considered being under Children’s labor age. This included working in a small industry, selling goods in the street, markets or shops, agriculture, and other casual jobs. Anxiety symptoms were measured by the Spence Children’s Anxiety Scale. The study found that anxiety scores were predicted by selling in the streets, working to help families, low family income, and lack of health insurance [48].

In summary, it was clear that Palestinian children are considered to be at higher risk to develop serious psychological distress due to living in areas under ongoing shelling, siege, and other acts of military violence. The previous studies covered different types of traumatic events that a child may have been exposed to in the particular circumstances of the occupied region. The findings indicated a significant proportion of Palestinian children experiencing serious psychological distress especially anxiety and PTSD. Several physiological symptoms were reported including insomnia, nightmares, sweating, and bedwetting.

#### Palestinian adolescents

Gaza Strip has been subjected to continuous traumatized events and blockade since the second Intifada (Uprising) in 2000. In this section, some studies were carried out to explore the response of adolescents to these events, for example, a randomized study conducted among 229 Palestinian adolescents from refugee camps of Rafah and Khan-Younis. The sample consisted of males (52.8%), and females (47.2), their ages ranged from 15 to 19 years. Participants were administered the following measures: The Posttraumatic Stress Disorder Interview (PTSD-I), and the Beck Anxiety Inventory (BAI). The study found that 68.9% of the sample was classified as having developed PTSD and 94.9% of the sample was classified as having severe anxiety levels [49].

Another study was conducted during the Second Intifada. Many adolescents were exposed to direct severe injury which left them with a permanent disability. The study was designed to assess the occurrence of Psychiatric disorders, in particular, PTSD and anxiety among Palestinian adolescents following intifada-related injuries. The sample consisted of 179 boys previously injured during the intifada and as a result, sustained a permanent disability. Approximately 76.5% of the injured victims qualified as having PTSD and with excess risk for chronic symptoms and comorbidity with other psychiatric disorders such as anxiety and depression. PTSD was positively associated with fatalism and negative coping among adolescents. Moreover, those adolescents with higher levels of anxiety and depression reported frequent use of negative coping strategies [50].

Additionally, a study regarding adolescents was a part of a larger survey conducted on a sample of Jewish and Arab-Palestinian school students in northern areas including the major city of Haifa which is inside 1948 occupied land. The study aimed to explore the influence of the second Lebanon War on adolescents’ Posttraumatic Stress Symptoms (PTS). The sample included 1800 Jewish and 2351 Arabs from a high-grade student. Although the study found exposure to war events had similar effects on both Arab and Jewish students, Arab-Palestinian reported higher PTS symptoms. One potential explanation may be that although they were exposed less than their Jewish peers, Arab adolescents felt much less secure, as they did not have shelters and protected areas, and only a few were evacuated from the danger zone. They feel less supported due to their experience of humiliation, discrimination, and injustice by the Israeli government [51].

Also, a study conducted among Palestinian adolescent’s cases aimed to advance the theory of chronic and traumatic stressors. The sample consisted of 438 Palestinian adolescents from the West Bank who had been exposed to several types of trauma. The ages of the sample were between “12–19”. The study included a measure for cumulative traumas that are based on the DBTF (Development-Based Trauma Framework) and other measures of anxiety and PTSD. The study showed that continuous traumatic stress was a significant predictor of mental health. They found that anxiety has an effect on decreased physical and mental health as well as on increased fear of death [52].

Similar to another study conducted randomly among (566) students to explore the perception of student psychological problems at An-Najah National University through the Al-Aqsa Intifada as a result of the Israeli occupation. The results of the study reveal that: the psychological effect was high among the students. The mean score was 61.2%. Most of the students were depressed and feeling anxious all of the time with a percent of 80.40%. On the other hand, the researchers pointed out the positive effect of the Al-Aqsa Intifada on the students and the Palestinian people from the student’s perception including (a) Feelings that they will have soon on the independent state of Palestine. (b) Feeling that the Intifada could help the whole world understand that the Israeli government doesn’t commit to the peace process, to the Oslo accord, and the Palestinians have rejected Israeli occupation [53].

In summary, the established studies mentioned above suggested a significant proportion of Palestinian adolescents living especially in the Gaza Strip and West Bank were the most vulnerable populations in the region and are more likely to experience psychological disturbances and report significant traumatic experiences. The results indicate those adolescents with higher levels of anxiety, depression, and PTSD reported frequent use of negative coping strategies.

#### Palestinian women

In this section, the following three studies were conducted and discussed to determine the anxiety level and PTS symptoms among Palestinian women. The first one: a descriptive study focused on infertility and its effects on Palestinian women’s mental health condition. It has come to assess psychological distress in infertile women living in the West Bank in Palestine. Researchers investigated the psychological distress among infertile women using the 90-R list of symptoms, a uniting tool to measure the status of current psychiatric symptoms. Results showed that infertile women appeared to complain of many psychological effects including a high level of anxiety and anxiety phobia [54].

The second descriptive study was conducted in (2016) in 9 refugee camps in the West Bank at UNRWA primary health care centers in three major cities (Nablus, Ramallah, and Hebron). The sample consisted of (327) pregnant women who were selected through random sampling. (GAD-7) used to measure the level of anxiety. The prevalence of anxiety was high. Pregnant women had a different degree of anxiety as follows: mild anxiety was 30.7%, moderate anxiety 17.5%, and severe anxiety was 12% [55].

Finally, Binge eating disorder (BED) and anxiety are deeply intertwined and often co-occur among females. One hundred fifty-four female undergraduate students from three different faculties of Polytechnic university- Hebron – West bank, Palestine were assessed. The screening for the presence of binge eating symptoms was done using BEDS-7. The study found half of the participants (50%) had binge eating symptoms in which binge eating disorder significantly correlated with psychosocial factors including a higher score of depression, stress, and anxiety [56].

In summary, the Palestinian women experienced psychological symptoms including a deferent level of anxiety associated with infertility, pregnancy,and BED.

#### Anxiety among patients with physical diseases

The following three studies were found and discussed the combination of anxiety level and physical diseases in Palestine. The first study was conducted to investigate the prevalence of PTSD and anxiety among children with cancer. The sample consisted of all children cases coming to be diagnosed and treated in the pediatric Oncology Unit, at El Nasser pediatric hospital in Gaza city. The sample included 23 males and 27 females. The results showed that 22% of children had partial PTSD and 18% had full criteria of PTSD, 62% of children had anxiety disorders [57].The second study was conducted among patients consecutively admitted to the cardiology and cardiac surgery departments of An-Najah National University Hospital, Arab-Specialized Hospital, Al Watani Hospital, and Nablus Specialized Hospital in the Northern West Bank city of Nablus. The sample consisted of a total of 1053 Patients. The age ranged 30–80. Interviews were conducted within 1 week of their admission to the hospital. Anxiety was more prevalent among females and less educated patients. Patients with anxiety symptoms reported poor social support and lower resilience [58].

The third study is a prospective, randomized, and controlled study that aimed at assessing the impact of preoperative education on the level of anxiety of patients undergoing abdominal surgery and postoperative pain. The study population consisted of adult men and women over 18 years old undergoing any type of elective abdominal surgery in governmental hospitals in the Nablus district – Palestine. The research showed a higher level of anxiety in the control group due to the lack of a structured educational program. It was clear that there was a significant relationship between the preoperative level of anxiety and the postoperative level of pain. It was clear that the level of pain decreased when the level of anxiety was lower. Preoperative anxiety can increase postoperative pain, the amount of analgesic that may be taken, as well as staying in the patient’s hospital. The study explored that preoperative education has been effective in reducing preoperative anxiety among patients who have undergone abdominal surgery, reducing postoperative pain, and improving vital signs. Moreover, they recommended that preoperative health education be included in routine care in preoperative preparations for surgical patients [59].

In summary, the Palestinian children experienced anxiety related to the health condition such as cancer, cardiac problems and who were undergoing elective abdominal surgery.

#### Gender and age differences

Some of the studies were found and discussed the relation between anxiety,and other multiple variables such as gender and age differences. A study aimed to detect the prevalence of behavioral and emotional problems among Palestinian children in the Gaza Strip. The sample consisted of 453 boys and 506 girls. The total number of schools selected was 42 UNWRA, 53 Government, and 2 Private schools. Teachers completed the Rutter scale B2 which consists of 26 items concerning child behavior. Factor analysis of the scale revealed the following three factors: antisocial behavior, anxiety, and school phobia. Boys and girls were compared in terms of the total score. The most frequently reported an emotional item was worrying, in addition to restlessness and poor concentration. The study indicated girls were less likely to be affected than boys [60]. The Palestinian girl had more educational items, peer component, and social skills, personal skills, spiritual beliefs, culture. This means girls were more protective in which they were more resilient [61].

In contrast, another study used a descriptive-analytic study to investigate the effect of traumatic events on children who were exposed to the Israeli military operation on the Gaza Strip in November 2012, and who lived in five localities of the Gaza Strip (north Gaza, Gaza, Middle area, Khan Younis, and Rafah area). The results indicated that 30.9% of children had an anxiety disorder. Anxiety was more in children living in camps and family monthly income less than $300. No differences in anxiety disorder between boys and girls who their age ranged between 9 and 16 years. The most common anxiety symptoms reported by children were: Other children are happier than me (71.7%), Others seem to do things easier than I can (81.3%), and I get nervous when things do not go the right way for me (64.3%). The results indicate Palestinian children used different ways of coping with stress and trauma, and common resilience items such as spiritual (religious) beliefs, sense of belonging, citizenship, and feeling safe when they were with their caregivers [61].

In summary, it seems from all the above-presented studies that anxiety disorders can exist among all Palestinian population. Children and adolescents were a particularly vulnerable target group. Trauma due to the war increased children’s psychological symptoms, including PTSD and anxiety. Such psychological problems were associated with traumatic experiences, and trauma decreases children’s resilience. Unfortunately, a few studies had explored the prevalence of anxiety and PTSD among Palestinian women and men. Moreover, anxiety disorders were associated with several physical conditions in the community such as cancer and cardiac.

## Discussion

In this paper, several studies were conducted mainly among children and adolescents. They have lived their entire lives under military occupation and with increasing life challenges. Researchers explored the prevalence to develop PTSD and anxiety disorders symptoms within these traumatized events [44–46, 49, 50, 52, 53]. While other complex sets of risk factors, including personality, genetics, and life events have significantly associated with developing these psychological disorders [3]. Anxiety disorders were the most common psychiatric disorders among children and adolescents with a median age of onset of 11 years [62]. Between the ages of 9 and 12 years, children can distinguish between conflicting emotions, and between others’ accidental and intentional behaviors. Moreover, during adolescence, social cognitions are enhanced further, together with the development of belief systems, hypothetical and abstract thinking, and self-evaluation of their thought process [63]. However, this can be achieved in normal situations. From the previously presented studies in Palestine, it was evident that most of the representative sample consisted of different school ages. This means that age may not be a significant contributor to the development of PTSD and anxiety. Moreover, the results may indicate that if the trauma is severe and the stress is severe, the majority of survivors especially children and adolescents may have PTSD, a high level of trauma, or persistent anxiety. Therefore, dysfunctions in their natural performance and development according to their age, are to be expected following exposure to such of these traumatic events and anxiety disorders.

As well as, The DSM-5 outlines the diagnostic criteria for PTSD as having exposure to the traumatic event, the presence of some specific symptoms, persistence avoidance of stimuli, negative alteration in cognition, mood, arousal, and reactivity associated with the traumatic events [42]. It was significant from the previously presented studies in Palestine that the researchers have revealed strong relationships between the rate and type of trauma exposure and the occurrence of PTSD [44–46, 49, 50, 52, 53].

On the other hand, it was well established that events do not need to be traumatic to have a great impact on the development of anxiety disorders and PTSD. Aversive experiences at the doctor and dentist, with animals and insects, with being trapped or lost, with injury, and with strangers, can all precede the development of clinically significant anxiety [64]. Palestinian adolescents especially boys who developed various and continuous levels of anxiety would have to overlap with multiple types of challenges. For example, Part of them leaves schools at an early age because they have many responsibilities such as supporting his family [47]. This was due to hunger or severe poverty that families face on the daily basis with a lack of an effective welfare governmental system.

Moreover, it is well-documented that females are more likely than males to develop an anxiety disorder with lifetime and past-year rates of anxiety disorders being 1.5–2 times higher among females than males [65]. Similar to the previous Jordanian study female adolescents were significantly more likely to have mental disorders than males [18]. But in the previously presented studies in Palestine, one study indicated boys more likely to be affected than girls [60]. While the other showed not [61]. Males are generally significantly less likely to seek and receive mental health services compared to females [66]. Some of them considering going to such of these services is a sign of weakness and most of them don’t want to appear like that, it is like a caveman mentality. This might be due to a lack of awareness and stigma attached to mental illness. Regarding research findings, gender-related anxiety disorders and PTSD were not clear and that’s why we need further studies considering the gender among Palestinian people.

Furthermore, participation in the workforce will expose women to extra pressure besides her usual responsibilities at home as a housekeeper [67]. Women are considered as the core of Palestinian society while the previously presented studies focused on adolescents and children [24]. However, the results showed a lack of studies discussed anxiety disorders levels among these struggle Palestinian women and all factors that cause anxiety disorders.

As well as, the psychological wellbeing of the mother during pregnancy also has been posited to play a role in the healthy development of the offspring [67]. Maternal anxiety may be associated with behaviors that hurt placenta functioning, blood flow, or nutritional supply to the developing fetus. Several prospective studies have shown that antenatal maternal anxiety was associated with emotional and behavioral problems in the offspring [68]. From the previously presented studies in Palestine, Pregnant women had a different degree of anxiety from mild 30.7% to severe 12% [55]. Besides, infertile women also appear to complain of many psychological effects including a high level of anxiety [54]. The treatment itself may evoke anxiety, and the unpredictable outcome of IVF treatment may induce a depressive mood [69].

Anxiety disorders are uniquely associated with several physical conditions in the community, and this comorbidity is significantly associated with poor quality of life and disability [70]. The presence of a physical illness, especially a life-threatening illness, such as coronary heart disease considered life-threatening comorbidity with anxiety disorders [71]. The presence of an anxiety disorder may increase the likelihood of physical illness through biological mechanisms (e.g. changes in the hypothalamic-pituitary axis system or alterations in autonomic nervous system activity) [7]. In combination with recent data demonstrating that anxiety disorders are risk factors for suicidal behavior [72]. However, from the previous results in Palestine [57–59], less is known about the extent to which anxiety disorders as well as its’ relation to poor quality of life. From our experience as health professionals and researchers in the field, this might be due to the absence of mental health care in general hospitals. Usually, the health care in the hospital uses the biological model and not using the biopsychosocial model or holistic health care approach.

Finally, some studies established the underlying structure of the genetic and environmental risk factors for anxiety disorders as similar between men and women [73].However, men were not mentioned or investigated in the found results. We can say, studies are absent discussed anxiety in men, and a lack of studies had discussed the anxiety level among women in particular during the Israeli military operations. As well as, women are considered an important part of society; this presents the importance of the role of social and family support in helping them deal with stress and trauma. The faith and religious practices have positive effects on the Islamic Palestinian population in reducing anxiety levels such as praying. It enhances their resilience and ability to recover from continuous adversities and traumas. In addition to “Sumud Culture” which was developed by Palestinians as a resilient response to the history of continuous traumas and a threat to their existent in their homeland [74]. The Standard treatments including Cognitive-behavioral therapy (CBT) and selective serotonin reuptake inhibitors (SSRIs) showed a significant reduction in average psychological symptoms for PTSD and anxiety disorders for specific vulnerable populations including children living in the occupied zones [7, 8] However, according to the literature results, there is a lack of studies that discussed the effectiveness of the treatment approach among patients who were diagnosed with anxiety disorders and PTSD.

Moreover, quantitative studies are more common in Palestine. This is because they are less costly than qualitative studies and less time-consuming. These quantitative studies may explain the extent to which Palestinians live under chronic intense pressures, but qualitative studies are still needed to be able to develop these findings further. It also seems that Palestinian researchers are not working collaboratively due to a lack of freedom to travel, speak, siege and other occupation practices. External research funds are also politically dependent, it also appears to be very little and increases when there is a high level of violence [24].

After considering this point regarding the research condition in Palestine, there is a need for drawing attention to the importance of providing a clear and updated database to deal with several aspects of scientific research. Also, it will enhance the collaboration between all researchers. As well as providing financial resources to train and support academics in the field of scientific research [75]. In the end, Palestinians facing complex challenges on the daily bases due to the Zionist occupation [32]. These challenges enhanced the whole Palestinian community to develop resilient responses to survive in face of ethnic cleansing policy against them^.^ More researches needed in the mental health field to support the existence of the Palestinian people [32, 74].

## Conclusion

Most of the studies were conducted during the Intifada period, so more representative of mental health status when there are high levels of violence in the surrounding environment. Anxiety disorders and PTSD were some of the most common mental disorders in Palestine. They develop from a complex set of risk factors, including genetics, personality, and life events. They are significantly associated with poor quality of life and disability. The research findings indicate that a significant proportion of Palestinian experiencing serious psychological distress especially anxiety and PTSD. Therefore, a mental health policy for Palestinians must deal with several challenges. Distinct barriers including inconsistent availability of medications, absence of multidisciplinary teamwork, insufficient specialists, fragmented mental health system, and occupation need to be addressed. Cognitive-behavioral therapy (CBT) showed a significant reduction in average psychological symptoms for PTSD and anxiety disorders for specific vulnerable populations especially children living in the occupied zones.

## Recommendations

The findings of PTSD and anxiety showed that there is a need for increasing the support for mental health services. As primary prevention, the occupation has to be considered as the main source of anxiety and other mental health disorders in Palestine. Besides, there is a need to implement a mental health care system through multidisciplinary work and raising awareness regarding the prevalence of mental disorders. Community mental health nurses can play a crucial role in enhancing mental health care in the local Palestinian community [32] Also, Humanitarian organizations should play a more positive role to protect the Palestinian community from the negative consequences of Israel’s occupation. Rather than a narrow medical perspective, there is an urgent need for the reconceptualization of Palestinian mental health using a public health approach, based on the broader frameworks of social justice, quality of life, human rights, and human security. Also, we recommend doing further studies regardless of types of anxiety disorders and the contributing factors with consideration of the effectiveness of the therapeutic-used approach. The interventions should take into consideration the Islamic culture which main source of resilience and Summed (steadfastness) among adults.

### Limitations

This paper has discussed anxiety disorders and PTSD in Palestine. Palestine is a state which seeks independence and freedom over the last 70 year [22]. Therefore a mental health system is facing specific challenges linked with occupation and political conflict. Mental disorders remain underreported, under-resourced, under-treated, and mental health services underfunded [32]. Besides, the research approach is still underdeveloped. The results indicated a lack of established literature studies regarding the topic. Due to this shortage, old reviews that are related to the topic were included. Also, there is a lack of studies that discussed different types of anxiety disorder e.g. (GAD) and the particular domains they affect throughout the life of both Palestinian men and women.

### Supplementary information

**Additional file 1.** PRISMA 2009 Checklist.

## Data Availability

This is an evidence synthesis study, all data is available from the primary research studies, or can be circulated from the corresponding author.

## Appendix A

**Table 1:** Search history for the Palestinian literature

Database/Search engine	Search #	Search terms/ Keywords/ combinations	Number of hits	Comments on your search or results (for instance comments on how you have combined terms)
PubMed https://www.ncbi.nlm.nih.gov/pubmed	1	Anxiety	227,089	
	2	Anxiety Disorder	147,410	
	3	Palestine	2619	
	1 + 2 + 3 Advanced search	((Palestine) AND anxiety disorder) AND anxiety AND PTSD	18	14
Science Direct	1	Anxiety Disorders and PTSD in Palestine	396	3
Google Scholar	1	Anxiety Disorders and PTSD in Palestine	3,850,000	6
An-Najah National University library	1	Anxiety disorders and PTSD	20	4

## Appendix B

**Table 2:** Attached studies characteristics

Title of the study	Author	Year	Aim	Methods	Measurements	Results	Section
Social adversities and anxiety disorders in the Gaza Strip 1998	Thabet AA, Vostanis P.	1998	To investigate the rate and nature of anxiety symptoms and disorders in children, and their relation to social adversities	237 children aged 9 to 13 years living in the Gaza Strip were selected randomly from 112 schools.	anxiety scale, Rutter scale	Children reported high rates of significant anxiety problems (21.5%) and teachers reported high rates of mental health problems in the children (43.4%) that would justify clinical assessment. Anxiety problems, particularly negative cognitions, increased with age and were significantly higher among girls. Low socioeconomic status (father unemployed or unskilled worker) was the strongest predictor of general mental health problems. Living in inner city areas or camps, both common among refugees, was strongly associated with anxiety problems.	Palestinian Children
Comorbidity of PTSD and depression among refugee children during war conflict 2004	Thabet AA, Abed Y, Vostanis P.	2004	To examine the prevalence and nature of comorbid post-traumatic stress reactions and depressive symptoms	403 children aged 9–15 years, who lived in four refugee camps,	Traumatic Events Checklist, the Child Post Traumatic Stress Reaction Index (CPTSD-RI), and the Short Mood and Feelings Questionnaire (MFQ).	The CPTSD-RI items whose frequency was significantly associated with total MFQ scores were: sleep disturbance, somatic complaints, constricted affect, impulse control, and difficulties in concentration.	Palestinian Children
The Effects of Chronic War Trauma among Palestinian Children 2008	Altawil M, Harrold, Altawil M, Asker A, Samara M, Harrold M.	2008	To explore the long-term effects of war and occupation on the Palestinian children in the Gaza Strip to explore the long-term effects of war and occupation on the Palestinian children in the Gaza Strip to explore the long-term effects of war and occupation on the Palestinian children in the Gaza Strip To explore the long-term effects of occupation on the Palestinian children in the Gaza Strip.	1137 children aged between 10 and 18 years were randomly selected from all parts of the Gaza Strip 1137 children aged between 10 and 18 years were randomly selected	Checklist of Traumatic Experiences (CTE), Symptoms of Post-Traumatic Stress Disorder Scale (PTSD) and Personality Assessment Questionnaire (PAQ).	The most prevalent types of trauma exposure for Palestinian children were as follows: 99% of children had suffered humiliation (either to themselves or a family member); 97% had been exposed to the sound of explosions/bombs; 85% had witnessed a martyr’s funeral and 84% had witnessed shelling by tanks, artillery, or military planes.	Palestinian Children
Exposure to war trauma and PTSD among parents and children in the Gaza strip 2008	Thabet AA, Abu Tawahina A, El Sarraj E, Vostanis P.	2008	To establish the relationship between ongoing war traumatic experiences, PTSD and anxiety symptoms in children, accounting for their parents’ equivalent mental health responses.	100 families, with 200 parents and 197 children aged 9–18 years, in areas under ongoing shelling and other acts of military violence.	(Gaza Traumatic Checklist), PTSD (Children’s Revised Impact of Events Scale, PTSD Checklist for parents), and anxiety (Revised Children’s Manifest Anxiety Scale, and Taylor Manifest Anxiety Scale for parents).	Both war trauma and parents’ emotional responses were significantly associated with children’s PTSD and anxiety symptoms.	Palestinian Children
Mental health problems among labour children in the Gaza Strip 2011	Thabet AA, Matar S, Carpintero A, Bankart J, Vostanis P.	2011	To establish the association between labour-related variables and mental health problems	780 children in labour (aged 9–18 years, mean 15.8) in the Gaza Strip.	Demographic checklist, the Strengths and Difficulties Questionnaire, the Spence Children’s Anxiety Scale and the Depression Self-rating Scale for Children.	Ratings of mental health problems were predicted by different factors, i.e. total difficulties scores by poor friendship relationships and lack of health insurance; anxiety scores by selling in the streets, working to help family, low family income and lack of health insurance; and depression scores by parents’ dissatisfaction with the job and longer working hours.	Palestinian Children
Post-traumatic stress disorder, depression, and anxiety among Gaza Strip adolescents in the wake of the second Uprising (Intifada) 2007	Elbedour S, Onwuegbuzie AJ, Ghannam J, Whitcome JA, Abu Hein F.	2007	To evaluate and describe the psychological effects of exposure of war-like circumstances on this population.	229 Palestinian adolescents from refugee camps their ages ranged from 15 to 19 years.	Posttraumatic Stress Disorder Interview (PTSD-I), and the Beck Anxiety Inventory (BAI).	Adolescents diagnosed with PTSD tended to be those who reported the highest levels of depression, anxiety, and positive reappraisal coping, and the lowest levels of seeking guidance and support coping.	Palestinian Adolescents
Post-traumatic stress and psychiatric disorders in Palestinian adolescents following intifada-related injuries 2008	Khamis V.	2008	To assess the occurrence of Psychiatric disorders, in particular, PTSD and anxiety among Palestinian adolescents following intifada-related injuries.	179 boys who were injured during Al-Aqsa intifada and as a result sustained a permanent physical disability.	Questionnaires were administered in an interview format	Approximately 76.5% of the injured victims qualify as having PTSD and that the disorder had a heterogeneous course, with excess risk for chronic symptoms and comorbidity with other psychiatric disorders such as anxiety and depression.	Palestinian Adolescents
High school students’ posttraumatic symptoms, substance abuse and involvement in violence in the aftermath of war 2012	Schiff M, Pat-Horenczyk R, Benbenishty R, Brom D, Baum N, Astor RA.	2012	To examine the effects of exposure to war events on adolescents’ Posttraumatic Stress Symptoms (PTS) and risk behaviors (substance use and involvement in school violence)	7th to 11th grade students from the north of Israel that included 4151 students: Jewish (54.4% boys) and Arab (41.5% boys).	Post Traumatic Stress Disorder (PTSD) scale	The Exposure to war events had similar effects on both Arab and Jewish students, Arab-Palestinian reported higher PTS symptoms	Palestinian Adolescents
Advances in Continuous Traumatic Stress Theory: Traumatogenic Dynamics and Consequences of Intergroup Conflict: The Palestinian Adolescents Case 2013	Kira IA, Ashby JS, Lewandowski L, Alawneh AWN, Mohanesh J, Odenat L.	2013	To advance the theory of chronic and traumatic stressors that have been identified as type III traumas in the trauma developmentally-based framework (DBTF) and use it to investigate the mental and physical health effects of such traumas on impacted individuals and groups.	438 adolescents from the West Bank who had been exposed to a number of types of trauma including chronic intergroup violence. Age ranged from 12 to 19	Post-traumatic stress disorder (PTSD), cumulative trauma related disorders (CTD), depression, anxiety, collective annihilation anxiety (AA), identity salience, and fear of death	Continuous traumatic stress was a significant predictor of mental health. They found that anxiety has an effect on decreased physical and mental health as well as on increased fear of death.	Palestinian Adolescents
The Perception of Student Psychological Problems at An-Najah National University through the Al-Aqsa Intifada as a Result of Israeli Occupation 2005	Assaf AM.	2005	To determine the most important psychological problems facing An-Najah National University students as a result of the Israeli aggression.	A random sample of (566) students	A scale of psychological problems was constructed and validated by the researcher and applied to the sample of the study	The psychological problem over all mean on students due to Israel aggression was (61.2%) which considered in terms of psychological effects is high	Palestinian Adolescents
Psychological Distress Among Infertile Women Attending Razan Center In West Bank In Palestine: Quantitative Study 2013	Katwsa L.	2013	To investigate the impacts of infertility on Palestinian women’s mental health status and to investigate the most prevalent psychological problems among infertile women.	88 women diagnosed with infertility	SCL.90-R (a self report measure of mental health symptomatology)	Infertile women in the current study have more psychological distress as represented through the 3 indices and 9 symptom dimensions of the SCL-90-R, than fertile women. The infertile women appeared to complain of many psychological effects including a high level of anxiety and anxiety phobia.	Palestinian Women
Anxiety and Depression, and their Associated Factors among pregnant women in Palestinian refugee camps - west bank	Iznait AA	2017	to find out the prevalence rate of depression and anxiety among pregnant women and the related associated factors during the period of study	(327) pregnant women who were selected through random sampling	(GAD-7) used to measure the level of anxiety, and depression (PHQ-9) Depression Scale.	The prevalence of anxiety was high. Pregnant women had a different degree of anxiety as follows: mild anxiety was 30.7%, moderate anxiety 17.5% and severe anxiety was 12%.	Palestinian Women
Binge eating symptoms prevalence and relationship with psychosocial factors among female undergraduate students at Palestine Polytechnic University: a cross-sectional study. 2019	Badrasawi MM, Zidan SJ.	2019	To examine the prevalence of binge eating symptoms and its relationship with selected variables (i.e. socio-demographics, nutritional status and dietary habits).	154 female undergraduate students, from three different faculties at Palestine Polytechnic University, participated in the study	The screening for presence of binge eating symptoms was done using BEDS-7.	Half of the participants (50%) had binge eating symptoms. No association between binge eating symptoms and socio-demographic variables was found. A significantly higher score on depression, stress and anxiety was found among binge eaters than non-binge eaters.	Palestinian Women
The Relationship between PTSD, Anxiety and Depression in Palestinian Children with Cancer and Mental Health of Mothers. 2017	Thabet A, Mansour M.	2017	To investigate the prevalence of PTSD, depression and anxiety among children with cancer and relationship to mother’s mental health.	50 children with their mothers was selected from oncology department at El Nasser paediatric hospital in Gaza city.	**A pre-designed socio-demographic sheet**	22% of children had partial PTSD and 18% had full criteria of PTSD, 62% of children had anxiety disorders	Palestinian Women
Depression and anxiety symptoms in cardiac patients: a cross-sectional hospital-based study in a Palestinian population 2019	Allabadi H, Alkaiyat A, Alkhayyat A, Hammoudi A, Odeh H, Shtayeh J, et al.	2019	To investigate the proportion of cardiac patients with depression and anxiety as well as factors associated with the presence of these symptoms in a Palestinian population.	The sample consisted of a total of 1053 Patients. The age ranged 30–80.	Cardiac Depression Scale (CDS) and the Depression Anxiety Stress Scale (DASS-42)	Symptoms of depression and anxiety were more prevalent among females and less educated patients. Factors independently associated with both depressive and anxiety symptoms were post-traumatic stress disorder symptoms, low level of self-esteem, high somatic symptoms, low physical and mental health component scores, active smoking, physical inactivity, and longer disease duration. Patients with depressive and anxiety symptoms also reported poor social support and lower resilience.	Anxiety among patients with physical diseases
The Impact of Preoperative Education on the Psychological and Physiological Aspects of Patients Undergoing Abdominal Surgery 2017	Thabet A, Mansour M.	2017	To assess the impact of preoperative education on the anxiety level of patients undergoing abdominal surgery and their postoperative pain.	Randomized and controlled among Adult men and women over 18 undergoing any type of elective abdominal surgery in governmental hospitals in the Nablus district.	Anxiety Inventory and the Visual Analog Scale (VAS) for pain scales	There was a significant reduction in the preoperative level of anxiety and postoperative level of pain among the patients who received the structured education program	Anxiety among patients with physical diseases
Epidemiology of child mental health problems in Gaza Strip 2001	Mousa Thabet AA, Vostanis P.	2001	To detect the prevalence of behavioral and emotional problems among Palestinian children.	The sample consisted of 453 boys and 506 girls. The total number of schools selected was 42 UNWRA, 53 Government and 2 Private schools.	Teachers completed the Rutter scale B2	The case incidence in boys was (54.5%), while in girls it was (46.5%).	Gender and age differences
Trauma, PTSD, Anxiety, and Resilience in Palestinian Children in the Gaza Strip.	Thabet A, Thabet S.	2015	To investigate types of traumatic events due to war on Gaza experienced by Palestinian adolescents in relation to PTSD and anxiety and coping strategies as mediating factor	One hundred fifty eight were boys (44.1%) and 200 were girls (55.9%)	The adolescents were interviewed by self -administrated questionnaire include sociodemographic scale, Gaza Traumatic Events Checklist, Spence Children’s Anxiety Scale, Post-Traumatic Stress Disorder according to DSM-IV scale, and Adolescent-Coping Orientation for Problem experiences Scale.	The results showed the mean total anxiety was 41.18, obsessive compulsive subscale was 8.90, generalized anxiety subscale was 4.46, social phobia was 6.99, separation anxiety was 6.16, physical injury fears was 5.48, and panic/Agoraphobia was 5.4. Girls had more anxiety problems than boys including all anxiety subscales. Regard PTSD, the study showed that 11.8% of adolescents reported no PTSD, 24.2% reported less than two clusters of symptoms, and 34.31% reported symptoms meeting criteria for partial PTSD, while 29.8% reported symptoms meeting criteria for full PTSD according to DSM-IV-TR. The results showed that girls reported more PTSD than boys	Gender and age differences

## Abbreviations

PTSD: Post Traumatic Stress Disorders
PA: Palestinian Authority
UNRWA: United Nations Relief and Work Agency for Palestinians
WHO: World Health Organization
NGOs: Non-governmental organizations
GCMHP: Gaza Community Mental Health Project
GAD: Generalized Anxiety Disorder
SAD: Social Anxiety Disorders
SP: Specific phobia
PD: Panic disorder
MENA: The Middle East and North Africa
SSRI: Selective serotonin reuptake inhibitors
TCAS: Tetracyclic antidepressant
